# Diphtheria

**Published:** 1883-11-20

**Authors:** Charles J. Burroughs

**Affiliations:** Georgia


### Southern Medical Record:

EDITORS:

T. S. POWELL, M.D. W. T. GOLDSMITH, M.D. R. C. WORD, M.D.

*R. C. WORD, M.D., Managing Editor.*

☞ All Communications and Letters on Business connected with the RECORD must be addressed to the Managing Editor.

VOL. XIII. ATLANTA, GA., NOVEMBER 20, 1883. No. 11

### ORIGINAL AND SELECTED ARTICLES.

### DIPHTHERIA.

BY CHARLES J. BURROUGHS, M.D., OF GEORGIA.

The subject under consideration is one upon which much has been written, but as each year we see more of it in our practice, we cannot be too familiar with all that pertains to it.

It has existed under many names, but that by which it is now recognized is a synonym of *diphtherite*, a word coined by Bretonneau from the Greek *diphthera*, a skin or parchment, and *itis*, implying inflammation.

It has been thought by some that its early history dates beyond the days of Hippocrates, but by others it is doubted if observations then referred really to diphtheria. The "Syriac Ulcer" mentioned by Aretans is considered as having more resemblance to this disease than any other of antiquity.

It was not until the 16th century that we find diphtheria fully recognized, and we are indebted to a French physician named Baillou for an accurate description of it. Other physicians, such as Tilla Real, Fontecha, Herrcra, Cortesius, Alaymus, Ghesi and John Fothergill, have written on diseases, nearly all of which can be credited to diphtheria.

In the year 1789 Dr. Samuel Bard, of Philadelphia, published a very interesting history of "An uncommon, and highly dangerous Distemper," which had recently proved fatal to many children in New York. Dr. Abercrombie wrote an accurate description of this affection in 1828. It prevailed as an epidemic in Edinburgh in 1826, and in Paris in 1853. We find it stated in one of our text-books that no deaths from diphtheria are reported as occurring in Philadelphia prior to 1860, but many cases are known to have been reported under the head of croup.

It is now, however. a disease we are often called upon to combat. It attacks the high and low. rich and poor, and whilst we most frequently find it among children, it is not uncommon among adults.

We see no partiality to any particular sex. To the seasons, it seems more to favor the fall and winter, more especially the former, judging from our experience, but we have met with it even in the heat of summer—having attended two cases since commencing this article, (July).

*Cause.*—Although the disease often arises in connection with bad drainage, foul habits and impure water supplv, we have seen it originate where we could not trace it to any of these causes especially. There is no doubt that too little attention is paid to proper rules of hygiene. If this were done there would be less liability to disease, but even where extra caution has been observed, by some means or other this disease has obtained a foothold.

It is remarkable how long the germs may remain dormant and then develop under certain conditions. Instances are recorded where the virus has remained latent for months, and in one case for three years, and then became active.

*Is the disease contagious ?*—As such it is considered by many of our best clinical observers, but the experience of others do not sustain their views. It is well, however, for the physician to act upon the affirmative side of this question when called upon to treat the disease. We have always endeavored to quarantine our patients, keeping other members of the family, particularly the younger children, whom we consider more susceptible to the poison, away from them ; but we have often found our commands disregarded—the well children mingling with the sick—and have seldom seen any evil result from it.

We may here state, in connection with this subject, that some remarkable cases of family susceptibility have been reported—one of which, a poor woman with three children of her own, had two others under her care. Her own children had the disease, one of whom died. The other children were not affected, though allowed to be constantly in the same room with the little patients. We have had a family under our care in which there were three cases in 1880. About eighteen months afterwards they were again visited by the disease, and five of the children were taken sick—one of whom was among those who had been previously attacked. Though in a part of the country that was thickly settled, there were no other cases in the immediate vicinity.

*Symptoms.*—When called to a case of diphtheria, in its incipiency, we find the neck swollen, with the parotid, submaxillary and lymphatic glands enlarged and tender. There is fever, the thermometer running as high sometimes as 103°. Upon examining the throat we discover the viscid white, or yellowish-white secretion, generally on one tonsil, which in a day or two extends to the other and then over the soft palate and pharynx. As the exudation extends the temperature in most cases subsides, though it may rise higher. The trachea and larynx are liable now to become diseased, thereby seriously affecting the life of the patient. The nasal passages may show the exudation—also any abrasions on the body. It is sometimes seen on the nipples of suckling women.

There is necessarily great pain and difficulty in deglutition, which is increased as the disease advances, the suffering of the patient at times being intense. Debility soon begins to be evident, the pulse becoming slow and weak, and the blood-poisoning each day becoming more and more manifest until death finally ends the struggle.

We have omitted, thus far, to speak of paralysis, cardiac thrombus and albuminuria, all of which a patient with diphtheria is liable to contend with. Albuminuria may appear at any time within the first few days, but has been delayed for two or three weeks. The paralysis and heart-clot are generally found at or near the termination of the case. Too often has it occurred that when to the fond parents of a beloved child over whom they had watched day and night, and to the physician who, like the helmsman, had carefully steered his frail bark through a long and perilous voyage, in which he knew not at what moment a storm might arise which he was illy able to contend against; but now, the voyage being almost ended, all could hope that their troubles were over, and anxiety no longer was visible on every countenance, one of these symptoms had presented itself. From paralysis we do not consider our patient free under two or three weeks, or even a month. We have discharged cases, their convalescence apparently complete, and have been called to them or have them returned to us with marked paralysis. We remember an interesting case of a boy whom we had attended and discharged, and some little while afterwards had him brought to our office with the statement that upon returning to school he had found it impossible to read, as the letters were all "jumbled up." He recovered in a short time upon treatment.

We have made no mention, thus far, of the croupous form of diphtheria, which Bartholow, in his able work says, "may begin as the ordinary catarrhal variety, and continue so without any indications of a departure from the usual course until the fourth or fifth day when it takes on a new character by its sudden development of high fever, increased tumefaction of the glands," etc. We have met with some of these cases, and their incipiency seem to be identical with the above description. The patient, for four or five days, would have the croupal cough, which increased and tightened as night came on, to be relieved by treatment until the following night when it would give symptoms of a repetition of the sufferings of the previous night.

In the treatment of this disease, we lay no claim to any originality, and will content ourselves by merely giving the formulæ we have generally found most advantageous to us. The following prescriptions we obtained some years ago from one of our journals, and deeming them good invariably use them—

| | | |
|---|---|---|
| No. 1.—℞. | Tinct. ferri chloridi........................ | ʒ i–iss, |
| | Glycerinum ........................ } Aqua pura........................ } | aa. ℥ i. |
| No. 2.—℞. | Potassæ chloras........................ | ʒ ss–i, |
| | Glycerinum........................ | ℥ ss, |
| | Aqua calcis........................ | ℥ iiss. |
| No. 3.—℞. | Acidum carbolicum........................ | gtt. xv, |
| | Aqua calcis........................ | ℥ vi. |

*Directions.*—Give one teaspoonful of No. 1 and No. 2, alternating every half hour, except at night, when allow to sleep one or two hours. Spray the throat with No. 3 for several minutes at a time whenever the above mixtures are given.

N. B.—Open the mouth wide. When the child is too young for this, omit it. When the nose is affected, syringe it out with warm or tepid salt water three or four times a day. Do not apply any brush or swab to the throat. Sometimes throw a teaspoonful of No. 1, with a syringe, directly against the affected surface of the throat. Give a plenty of cold milk, and give frequently; add limewater occasionally ; also give beef tea. This would be our general treatment, save that we watch our thermometer very closely, and if there is any fluctuation in it put in quinine *pro re nata*. If we are called early when there is much fever, we find the following prescription produce a most desirable effect—

| | | |
|---|---|---|
| ℞. | Tinct. gelsemium.......................<br>Tinct. aconite rad...................... | aa. gtt. xvi, |
| | Spiritus ætheris nitrosi.................... | ʒ ss, |
| | Aqua pura................................ | ℥ ii. |

Of this we give a teaspoonful every half hour or hour, according to the age of the child, until we find the temperature is on the decline. We give quinine three times a day for its tonic effect, and nourish as best we can with egg-nogg, milk-punch, chicken soup, etc. A warm flax-seed meal poultice we direct to be applied to the throat during the day and at night if the patient's sufferings are such as to prevent his sleeping. Should he rest well at night we remove the poultice and apply a thick and wide flannel collar fitting tightly. We find that sponging the body with equal parts of alcohol and tepid water two or three times a day tends greatly toward strengthening our patients.

During convalescence it is our custom to put our patients on the following:

| | | |
|---|---|---|
| ℞. | Chlo. potassa.................................. | ʒ i, |
| | Tinct. fer. chlo................................ | ʒ ii, |
| | Glycerine ...................................... | ℥ i, |
| | Aqua.......................................... | ℥ vi. |

M. Sig. Dose: one teaspoonful.

We give it for the first few days every three or four hours, afterwards three times a day, and keep it up for some time, say two or three weeks, as the blood-poisoning leaves an anæmia, from which recovery is slow, and we deem that this will tend greatly toward warding off paralysis, which may make its appearance during this time.

We are constrained to differ with our professional brethren who advocate so strongly a mercureal cathartic in the commencement of this disease. We do not consider a "scavenger," viz: calomel, jalap and scammony, as a necessary adjuvant. Our experience as far as producing satisfactory results, has been very different from those who found that "the course of the disease was certainly cut short" by these means. We attended a case of a child some time ago to whom ten grains of calomel had been given previous to our arrival. The *course of the disease was certainly cut short*, but not as we would have desired, as the patient died. We are willing to admit that the bowels must do their duty in eliminating the poison from the system, and we make use of them for this purpose, but we consider a mild cathartic occasionally given is sufficient. We try to give as little medicine of a debilitating nature, as possible, through the whole course of the disease, but strive rather to keep up the strength of our patients, and by using such treatment as we have given, we believe we have met with as favorable results as could be obtained from any other course.

